# Genetic Association and Clinical Relevance of TNFSF13B/BAFF and PADI4 Polymorphisms in ANCA-Associated Vasculitis: A Case–Control Study with Genetic Model Analysis in Guangxi Population

**DOI:** 10.3390/genes17060710

**Published:** 2026-06-20

**Authors:** Jiafu Lu, Simei Huang, Shuwen Wei, Chao Xue

**Affiliations:** The Second Affiliated Hospital of Guangxi Medical University, Nanning 530007, China; 202321312@sr.gxmu.edu.cn (J.L.); 2023121293@sr.gxmu.edu.cn (S.H.); 2023121311@sr.gxmu.edu.cn (S.W.)

**Keywords:** ANCA-associated vasculitis (AAV), *TNFSF13B/BAFF*, *PADI4*, single nucleotide polymorphism (SNP), genetic susceptibility, machine learning

## Abstract

**Objective:** *TNFSF13B*, which encodes B-cell-activating factor (*BAFF*) and peptidylarginine deiminase 4 (*PADI4*), plays crucial roles in the pathogenesis of ANCA-associated vasculitis (AAV). This study investigated the associations of single-nucleotide polymorphisms (SNPs) in TNFSF13B/BAFF and *PADI4* genes with AAV susceptibility, clinical phenotypes, and disease activity in a Guangxi Chinese population. **Methods:** A case–control study included 324 AAV patients and 324 healthy controls. After propensity score matching (201 pairs), genomic DNA was genotyped for *TNFSF13B/BAFF* rs3759467 (formerly rs386492354) and rs1041569, and *PADI4* rs11203366 and rs874881 using multiplex PCR and high-throughput sequencing. Genetic associations were analyzed via logistic regression, subgroup, haplotype, and clinical correlation analyses. For each of the four SNPs separately, machine learning models (logistic regression, SVM, Random Forest, XGBoost) were built and evaluated via 5-fold cross-validation. No formal adjustment for multiple comparisons was applied due to the exploratory nature of this study. **Results:** For *TNFSF13B/BAFF*, the rs3759467 C allele was protective (dominant model OR = 0.60, *p* = 0.011; log-additive OR = 0.71, *p* = 0.020; CA haplotype OR = 0.71, *p* = 0.019), while the rs1041569 T allele was a risk factor (dominant model OR = 1.70, *p* = 0.016). Subgroup analysis revealed stronger protective effects of rs3759467 in females, Han ethnicity, and MPA patients, and stronger risk effects of rs1041569 in Han ethnicity and MPA patients. Haplotype CA was protective (OR = 0.71, *p* = 0.019), and TT was risk-associated (OR = 1.55, *p* = 0.017). Both *TNFSF13B/BAFF* SNPs were associated with rash and hemoptysis incidence (*p* < 0.05). rs1041569 was also associated with RBC (red blood cell) count and HB (hemoglobin) levels (*p* < 0.05). For *PADI4*, rs11203366 and rs874881 showed no association with AAV susceptibility (all *p* > 0.05). However, their genotypes were associated with disease activity (BVAS, Birmingham Vasculitis Activity Score), RBC count, and HB levels (*p* < 0.05). Although machine learning was applied to explore predictive patterns, its performance was suboptimal (AUC < 0.6), indicating limited clinical applicability. Accordingly, the primary findings rely on the genetic model analysis, and the machine learning results should not be overinterpreted as clinically actionable. SHAP analysis indicated that risk-associated genotypes contributed most to model predictions. **Conclusions:** *TNFSF13B/BAFF* gene polymorphisms rs3759467 and rs1041569 were associated with AAV susceptibility in this Guangxi cohort, influencing clinical manifestations like rash, hemoptysis, and anemia severity. *PADI4* polymorphisms rs11203366 and rs874881 are not associated with susceptibility but may correlate with disease activity and hematological parameters. These findings highlight the ethnic and clinical subtype specificity of genetic influences in AAV. Due to the lack of external validation, these findings are exploratory and require replication.

## 1. Introduction

ANCA-associated vasculitis (AAV) is a group of severe autoimmune diseases characterized by necrotizing small-vessel inflammation, leading to high mortality and morbidity [1]. Genetic factors are believed to contribute significantly to its pathogenesis [2,3]. *TNFSF13B/BAFF*, a key cytokine for B-cell survival and antibody production, is overexpressed in active AAV and correlates with ANCA titers [4]. Moreover, B-cell-targeted therapy with rituximab (anti-CD20 monoclonal antibody) induces remission and improves renal function in AAV, confirming the central role of B cells in AAV pathogenesis. As a key regulator of B-cell survival and differentiation, *TNFSF13B/BAFF* may contribute to B-cell dysregulation and autoantibody production in AAV [5,6]. Peptidylarginine deiminase 4 (*PADI4*) catalyzes protein citrullination, essential for neutrophil extracellular trap (NET) formation, a core process in AAV vascular injury [7]. In lupus nephritis, *PADI4* is involved in the production of autoantibodies and deposition of immune complexes, exacerbating renal inflammation [8]. While polymorphisms in these genes are linked to other autoimmune diseases like rheumatoid arthritis and Sjögren’s syndrome [9], their role in AAV, particularly in Chinese populations, remains unclear. This study aimed to explore the associations of specific single-nucleotide polymorphisms (SNPs) in the *TNFSF13B/BAFF* and *PADI4* genes with disease susceptibility, clinical phenotypes and disease activity of AAV among the population in Guangxi, using conventional statistical approaches, genetic model analysis and machine learning algorithms.

## 2. Materials and Methods

### 2.1. Study Population

This case–control study enrolled 324 AAV patients and 324 healthy controls. AAV patients were recruited from the Department of Nephrology, The Second Affiliated Hospital of Guangxi Medical University (formerly the West Branch of the First Affiliated Hospital), between January 2006 and June 2025. Healthy controls were volunteers undergoing routine health examinations at the same hospital during the same period (2006–2025) as the AAV patients. Inclusion criteria were: (1) diagnosis of AAV according to the 2012 revised Chapel Hill Consensus Conference criteria; (2) registered residence in Guangxi; (3) age ≥18 years at enrollment; and (4) no blood relationship with other participants. Exclusion criteria included secondary vasculitis (e.g., associated with rheumatoid arthritis, systemic lupus erythematosus, undifferentiated connective tissue disease, infections, Henoch–Schönlein purpura, or malignancy), coexisting malignancy, severe multiple organ failure, serious hereditary diseases, or refusal to participate. Healthy controls were volunteers undergoing routine health examinations at the same hospital during the same period. Inclusion criteria were: no evidence of AAV or related conditions, Guangxi registered residence, age- and sex-matched to the AAV group, and no blood relationship with other participants. Individuals with autoimmune diseases, hereditary disorders, or major medical history were excluded. The study was approved by the Ethics Committee of the Second Affiliated Hospital of Guangxi Medical University. Written informed consent was obtained from all participants.

### 2.2. Clinical Data Collection

Clinical data were collected using a standardized case report form from the hospital’s electronic medical record and laboratory information systems. All clinical and laboratory variables were collected at the time of AAV diagnosis, prior to any immunosuppressive treatment. Collected information included: (1) baseline demographic data (sex, age, ethnicity, and medical history) for both groups; and (2) AAV-specific data, including clinical manifestations (e.g., rash, hemoptysis), laboratory parameters (blood routine, urinary albumin, Scr (serum creatinine), CRP (C-reactive protein), renal pathologic classification, and disease activity assessed by the BVAS (Birmingham Vasculitis Activity Score, BVAS).

### 2.3. SNP Selection

SNP selection was based on sequencing data from the 1000 Genomes Project (GRCh37, https://grch37.ensembl.org/, accessed on 13 May 2026) analyzed with Haploview 4.2 software. Selection criteria included: minor allele frequency (MAF) ≥ 0.05, Hardy–Weinberg equilibrium (HWE) *p* > 0.05 in controls, and prior reported associations with autoimmune diseases. SNP functional annotations were obtained from NCBI dbSNP (https://www.ncbi.nlm.nih.gov/snp/, accessed on 13 May 2026). Ultimately, two *TNFSF13B/BAFF* SNPs (rs3759467 and rs1041569) and two *PADI4* SNPs (rs11203366 and rs874881) were selected. This study used a candidate-gene and candidate-SNP strategy rather than comprehensive gene-wide tag-SNP mapping. *TNFSF13B/BAFF* and *PADI4* were selected because of their biological relevance to B-cell activation, autoantibody production, citrullination, and NETosis in AAV. The four SNPs were prioritized according to East Asian allele frequency, previous evidence in autoimmune diseases, and potential biological or functional relevance.

### 2.4. Genotyping

Peripheral venous blood (5 mL) was collected into EDTA tubes. Genomic DNA was extracted using a Blood DNA Kit (Tiangen Biotech, Beijing, China). DNA samples with an A260/A280 ratio of 1.7–1.9 and a concentration >25 ng/μL were included and stored at −80 °C. Genotyping was performed by Shanghai Sangon Biotech Co., Ltd. (Shanghai, China) using multiplex polymerase chain reaction (PCR) combined with high-throughput sequencing. All samples were anonymized and assigned random identification numbers before genotyping. Laboratory personnel were blinded to case/control status and clinical data.

### 2.5. Statistical Analysis

Statistical analyses were conducted using SPSS 27.0, SNPstats online tool, and Haploview software. All tests were two-sided, with *p* < 0.05 considered statistically significant.

#### 2.5.1. Propensity Score Matching and Baseline Comparison

To minimize baseline confounding, propensity score matching (PSM) was performed. A logistic regression model was constructed with AAV status as the dependent variable and age, sex, and ethnicity as covariates. One-to-one nearest-neighbor matching with a caliper of 0.02 yielded 201 matched pairs. Matching quality was assessed by standardized mean differences < 10% for all covariates. Continuous variables were expressed as mean ± standard deviation or median (interquartile range) and compared using an independent *t*-test or the Mann–Whitney U test. Categorical variables were presented as *n* (%) and compared using chi-square or Fisher’s exact test.

#### 2.5.2. Association Analysis of Genetic Variants

Genotype distributions in controls were tested for HWE using chi-square tests (*p* > 0.05). Associations between SNPs and AAV risk were evaluated using unconditional logistic regression to calculate odds ratios (ORs) and 95% confidence intervals (CIs) under multiple genetic models (codominant, dominant, recessive, overdominant, and log-additive) via SNPstats. Genotype encoding: SNPs were encoded additively as 0, 1, or 2 minor alleles. Data splitting: five-fold cross-validation with a fixed random seed, no separate test set. Hyperparameter tuning: Default algorithm parameters were used (no extensive tuning). Nested cross-validation: Not performed because no hyperparameter tuning was conducted. Haplotype analysis and linkage disequilibrium (LD) were performed with Haploview. Subgroup analyses were conducted by sex, ethnicity, and clinical subtype.

#### 2.5.3. Missing Data Handling

Missing clinical data were handled by multiple imputations using the fully conditional specification method in SPSS, generating five imputed datasets that included all analysis variables. The dataset with the highest Cronbach’s alpha score (excluding the original) was used for subsequent clinical parameter analyses.

### 2.6. Machine Learning–Based Analysis

Four machine learning algorithms were employed to explore the relationship between SNP genotypes and disease status and to build predictive models (Separate models were built for each SNP individually, using only that SNP’s genotype as the input feature): logistic regression (LR), Support Vector Machine (SVM with radial basis function kernel), Random Forest (RF), and Extreme Gradient Boosting (XGBoost). Model performance was evaluated using 5-fold cross-validation with a fixed random seed for reproducibility. In each fold, four subsets served as the training set and one as the validation set. Performance metrics included Accuracy (ACC), Precision (PRE), Recall (REC), F1-Score, and Area Under the Receiver Operating Characteristic Curve (AUC). The average values across five folds were reported. Model interpretability was assessed using SHAP (SHapley Additive exPlanations) values to quantify the contribution and direction of each genotype feature to predictions. Models were built primarily on the matched cohort (201 pairs) and additionally explored using the full dataset (324 AAV cases and 324 controls) for *TNFSF13B/BAFF* rs3759467, *PADI4* rs11203366, and rs874881 genotypes.

## 3. Results

### 3.1. Baseline Characteristics

After matching, the 201 AAV patients and 201 controls were balanced for age, sex, and ethnicity (all *p* > 0.05), Table 1. Functional annotation and selection rationale of candidate SNPs in *TNFSF13B/BAFF* and *PADI4*, Table S1.

### 3.2. Genetic Association Analysis

*TNFSF13B/BAFF* SNPs: rs3759467 genotype and allele distributions differed significantly between groups (*p* = 0.038, 0.024), Table 2. The C allele was protective (dominant model OR = 0.60; log-additive OR = 0.71), Table 2. rs1041569 allele frequency differed significantly (*p* = 0.021), Table 2, with the T allele being a risk factor (dominant model OR = 1.70; log-additive OR = 1.59), Table 3. Subgroup analysis showed the protective effect of rs3759467 was more pronounced in females (dominant OR = 0.52, *p* = 0.014), Han ethnicity (OR = 0.58, *p* = 0.029), and MPA patients (OR = 0.56, *p* = 0.0075), Table 4. The risk effect of rs1041569 was stronger in Han ethnicity (OR = 1.76, *p* = 0.035) and MPA patients (OR = 1.75, *p* = 0.015), Table 4. Haplotype analysis revealed CA as protective (OR = 0.71, *p* = 0.019) and TT as risk-associated (OR = 1.55, *p* = 0.017), Table 5. *PADI4* SNPs: rs11203366 and rs874881 showed no significant differences in genotype or allele frequencies between groups (all *p* > 0.05), Table 6. No significant associations were found in any genetic model or subgroup analysis. Strong linkage disequilibrium existed between them, but the formed haplotypes were not associated with AAV risk.

### 3.3. Association with Clinical Parameters

*TNFSF13B/BAFF* SNPs: Genotypes of both rs3759467 and rs1041569 were associated with the incidence of rash and hemoptysis (*p* < 0.05). rs1041569 genotypes were also associated with RBC count and hemoglobin levels (*p* < 0.05), with the TT genotype showing the most severe anemia. No significant associations were found with BVAS, WBC, PLT, CRP, urinary albumin, or creatinine. Table 7. *PADI4* SNPs: rs11203366 and rs874881 genotypes were associated with BVAS (*p* = 0.029, 0.032), RBC count, and hemoglobin levels (both *p* < 0.001), Table 8.

### 3.4. Machine Learning Analysis

**Figure 1:**
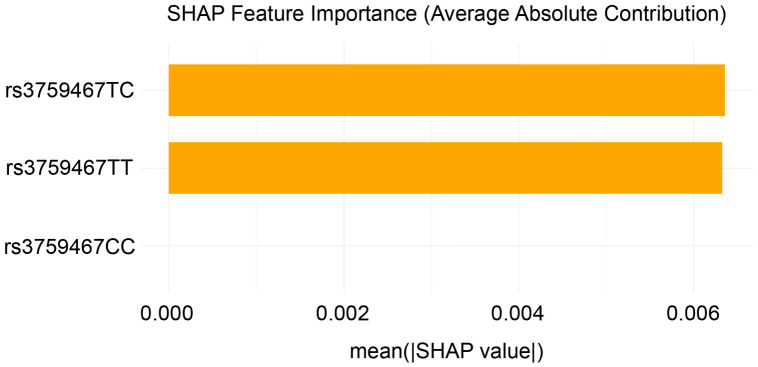
SHAP values for rs3759467 (formerly rs386492354). Genotypes carrying the T allele (TT and TC) showed higher contribution scores, consistent with the protective effect of the C allele.

**Figure 2:**
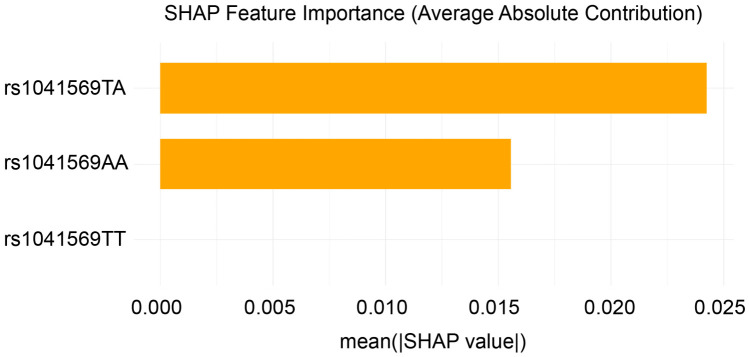
SHAP values of the XGBoost model in the rs1041569 group.

**Figure 3:**
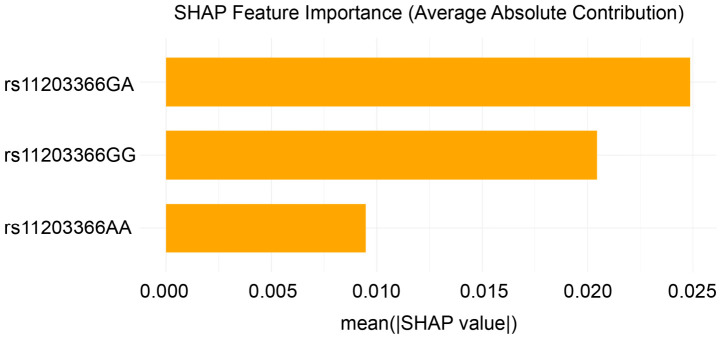
SHAP values of the XGBoost model in the rs11203366 group.

**Figure 4:**
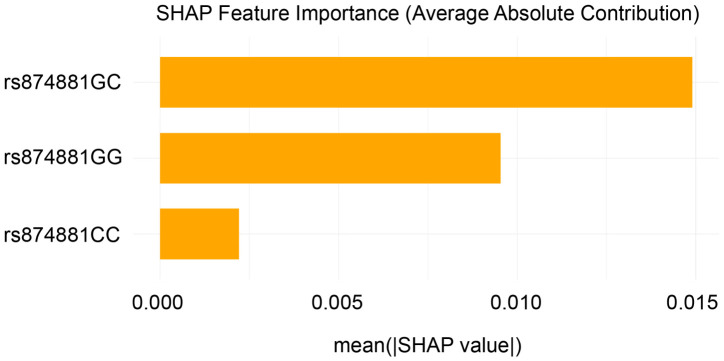
SHAP values of the XGBoost model for rs874881 subgroups.

For all SNPs, the machine learning models demonstrated limited discriminative ability. The highest average AUCs were 0.5362 (XGBoost for *TNFSF13B/BAFF* rs3759467), 0.5349 (XGBoost for *TNFSF13B/BAFF* rs1041569), 0.5225 (XGBoost for *PADI4* rs11203366), and 0.5395 (XGBoost for *PADI4* rs874881), all below 0.6, Table 9a–d. SHAP analysis indicated that genotypes carrying the T allele (TT and TC for rs3759467) contributed most to model predictions. However, given the near-random performance of the models (AUC < 0.6), these SHAP values should not be interpreted as biologically meaningful, Figure 1, Figure 2, Figure 3 and Figure 4.

## 4. Discussion

As a member of the tumor necrosis factor (TNF) superfamily, *TNFSF13B/BAFF* serves as an essential survival factor for transitional and mature B cells [10]. *TNFSF13B/BAFF* overexpression expands autoreactive B-cell compartments and induces systemic autoimmunity in murine models [11], mirroring the disease-promoting effect of the rs1041569-T risk allele identified in our cohort, Table 3. Autoantibody levels and synovitis in early rheumatoid arthritis have been associated with increased *BAFF* concentrations in a subset of patients [12]. Chronic inflammation is the core pathological foundation of anemia of chronic disease. It induces anemia through multiple mechanisms, including disrupting iron homeostasis, suppressing erythropoietin activity, and reducing erythrocyte survival time [13]. As shown in Table 7, carriers of the rs1041569-TT genotype presented with more severe anemia, accompanied by decreased hemoglobin-to-red blood cell ratio (*p* < 0.05), which was consistent with findings reported in the previous literature. However, given the small number of TT homozygotes (n = 11), this finding is preliminary and requires validation in an independent cohort with a larger sample size. In primary Sjögren’s syndrome (pSS), one of the autoimmune disorders, Zheng A et al. confirmed that the SNP rs12583006 in the *TNFSF13B/BAFF* gene is significantly associated with disease risk in patients with pSS. [14]. Further subgroup analysis of this study indicated that the *TNFSF13B/BAFF* gene rs3759467 variants conferred a more remarkable protective effect in female Han patients diagnosed with an MPA subtype. Meanwhile, the rs1041569 polymorphism showed an enhanced risk effect among Guangxi Han patients with MPA. The subgroup analysis based on AAV clinical subtype (MPA vs. non-MPA) is limited by the very small sample size of the non-MPA subgroup (n = 15). Consequently, comparisons between MPA and non-MPA are underpowered, and the observed differences (or lack thereof) should be considered exploratory. Independent replication in larger cohorts with balanced subtype representation is required. AAV is regarded as a complex disorder, and its onset is attributable to the interaction between numerous weak-effect genetic variations and environmental factors. This study provides novel evidence that *TNFSF13B/BAFF* gene polymorphisms are associated with AAV susceptibility in a Guangxi Chinese population, with effects modulated by sex, ethnicity, and clinical subtype (MPA). The contrasting roles of the C allele (protective) and T allele (risk) underscore the complexity of *TNFSF13B/BAFF* pathway regulation in AAV. Their associations with specific clinical manifestations (rash, hemoptysis, anemia) suggest that genetic background may shape disease phenotype, possibly via influencing inflammation severity or B-cell/antibody activity. However, the associations with rash and hemoptysis are based on relatively small event numbers; therefore, these findings should be considered exploratory and hypothesis-generating. Independent replication in larger cohorts using methods appropriate for sparse data (e.g., exact logistic regression) is warranted.

Conversely, *PADI4* polymorphisms rs11203366 and rs874881 did not confer susceptibility, distinguishing AAV from diseases like RA where *PADI4* is a strong genetic factor. Clinical studies provide direct evidence for this: serum *PADI4* levels are significantly elevated in patients with active AAV disease and positively correlated with disease activity, suggesting that *PADI4* plays a role in the pathogenesis of AAV [15]. Certain *PADI4* haplotypes improve mRNA stability and subsequently facilitate protein citrullination. This cascade is critical for the generation of anti-citrullinated protein antibodies and represents a central immunopathological mechanism in RA development [16,17]. Massarenti et al. reported that carriers of rs11203366, rs874881 and other polymorphisms have a higher prevalence of lupus nephritis and hypertension [18]. The primary function of *PADI4* is to modulate neutrophil extracellular trap formation in inflammation via catalysis of protein citrullination [19,20]. Sustained inflammation inhibits bone marrow hematopoiesis, resulting in decreased red blood cell production and impaired hemoglobin synthesis [21,22]. The observed associations between *PADI4* genotypes and hemoglobin/erythrocyte levels are consistent with a potential role of chronic inflammation, but this interpretation is hypothesis-generating and lacks direct evidence from our data. This mechanistic implication is further corroborated by the significant correlational observations presented in Table 8. Further analysis indicated that the homozygous genotypes AA/GG at rs11203366 and GG/CC at rs874881 were significantly correlated with elevated BVAS scores and increased disease activity. This is mechanistically consistent with our finding that rs11203366 and rs874881 are associated with disease activity and anemia in AAV, suggesting that *PADI4* may predominantly influence disease severity rather than disease susceptibility after disease onset. Given the aforementioned correlations and molecular mechanisms, their association with disease activity and anemia indicators implies a role in modulating disease severity or inflammation after onset, potentially through NETosis regulation. Considering that anemia of chronic disease is highly prevalent among patients with AAV, its central pathogenesis is driven by inflammatory cytokines such as IL-6, which induce disordered iron metabolism and erythropoietin resistance [23]. The results of the present study suggest that the GA genotype of rs11203366 and the GC genotype of rs874881 are associated with higher red blood cell count and hemoglobin level.

The limited predictive power of machine learning models using these SNPs alone highlights that AAV is a complex disease influenced by numerous genetic and environmental factors. SHAP values were calculated for exploratory feature importance description, but due to low model performance, no causal or biological claims are drawn. A limitation of this study is the lack of an a priori sample size calculation, which was not feasible given the exploratory nature of the analyses using an existing dataset.

In conclusion, *TNFSF13B/BAFF* rs3759467 and rs1041569 polymorphisms showed associations with AAV susceptibility in this Guangxi cohort, but external validation is needed. *PADI4* rs11203366 and rs874881 were not susceptibility factors for AAV in this cohort, but they were associated with disease activity and anemia parameters, suggesting a potential role in disease severity. These findings emphasize the importance of population-specific and subtype-stratified genetic studies in AAV. Because only two candidate SNPs per gene were included, this study does not represent comprehensive genetic coverage of *TNFSF13B/BAFF* or *PADI4*. The findings should therefore be interpreted as exploratory candidate-SNP associations and require replication in larger cohorts and functional validation. A major limitation is the lack of an independent replication cohort. Multiple comparisons may have inflated type I error; therefore, these findings should be interpreted as hypothesis-generating and need validation in future studies. The long recruitment period (2006–2025, nearly 20 years) is a limitation of this study, highlighting the need for future prospective studies with shorter, well-defined recruitment windows to validate the observed associations.

## Figures and Tables

**Table 1:** Demographic characteristics before and after matching.

Variable	Before PSM			After PSM		
	AAV (n = 324)	Control (n = 324)	*p*	AAV (n = 201)	Control (n = 201)	*p*
Sex			0.575			0.611
Male	126	133		83	78	
Female	198	191		118	123	
Nation			<0.001			0.747
**Variable**	**Before PSM**			**After PSM**		
	**AAV (n = 324)**	**Control (n = 324)**	***p***	**AAV (n = 201)**	**Control (n = 201)**	***p***
Zhuang ethnic	125	85		64	61	
Han ethnic	199	239		137	140	
Age			<0.001			0.307
(Year)	59.00 (48.25, 67.00)	45.00 (36.00, 53.00)		54.00 (40.50, 61.00)	51.00 (42.00, 57.50)	

**Table 2:** The allelic and genotypic distributions of two SNP loci in the *TNFSF13B/BAFF* gene between the AAV group and the control group.

SNP		Genotype			Allele	OR (95% CI)
rs3759467	TT	TC	CC	T	C	
AAV	93 (46.3%)	85 (42.3%)	23 (11.4%)	271 (67.4%)	131 (32.6%)	0.710 (0.530–0.947)
Control	68 (33.8%)	103 (51.2%)	30 (14.9%)	239 (59.5%)	163 (40.5%)	
*p*		0.038		0.024		
rs1041569	TT	TA	AA	T	A	
AAV	7 (3.5%)	64 (31.80%)	130 (64.7%)	78 (19.4%)	324 (80.6%)	1.586 (1.056–2.386)
Control	4 (2%)	45 (22.4%)	152 (75.6%)	53 (13.2%)	349 (86.8%)	
*p*		0.054		0.021		

**Table 3:** Association of *TNFSF13B/BAFF* gene polymorphism with AAV susceptibility under different genetic models.

SNP	Model	Genotype	Control	AAV	OR (95% CI)	*p*
rs3759467	Codominant	TT	68 (33.8%)	93 (46.3%)	1	0.040
		TC	103 (51.2%)	85 (42.3%)	0.60 (0.39–0.92)	
	Dominant	CC	30 (14.9%)	23 (11.4%)	0.57 (0.30–1.06)	
		TT	68 (33.8%)	93 (46.3%)	1	0.011
		TC-CC	133 (66.2%)	108 (53.7%)	0.60 (0.40–0.89)	
rs1041569	Log-additive	---	---	---	0.71 (0.53–0.95)	0.020
	Codominant	AA	152 (75.6%)	130 (64.7%)	1	0.050
		TA	45 (22.4%)	64 (31.8%)	1.67 (1.06–2.61)	
	Dominant	TT	4 (2%)	7 (3.5%)	2.11 (0.60–7.39)	
		AA	152 (75.6%)	130 (64.7%)	1	0.016
	Overdominant	TA-TT	49 (24.4%)	71 (35.3%)	1.70 (1.10–2.62)	0.033
		AA-TT	156 (77.6%)	137 (68.2%)	1	
		TA	45 (22.4%)	64 (31.8%)	1.62 (1.04–2.53)	

**Table 4:** Subgroup analysis of the association between *TNFSF13B/BAFF* rs3759467 polymorphisms and AAV susceptibility.

Group	Model	Genotype	Control	AAV	OR (95% CI)	*p*	Group	Control	AAV	OR (95% CI)	*p*
Female	Codominant	TT	38 (30.9%)	54 (45.8%)	1	0.035	Male	30 (38.5%)	38 (46.3%)	1	0.560
		TC	63 (51.2%)	51 (43.2%)	0.56 (0.32–0.98)			40 (51.3%)	34 (41.5%)	0.70 (0.36–1.37)	
		CC	22 (17.9%)	13 (11%)	0.40 (0.18–0.90)			8 (10.2%)	10 (12.2%)	0.96 (0.33–2.76)	
	Dominant	TT	38 (30.9%)	54 (45.8%)	1	0.014		30 (38.5%)	38 (46.3%)	1	0.370
		TC-CC	85 (69.1%)	64 (54.2%)	0.52 (0.30–0.88)			48 (61.5%)	44 (53.7%)	0.75 (0.39–1.41)	
Han ethnic	Log-additive	---	---	---	0.61 (0.42–0.90)	0.011		---	---	0.88 (0.55–1.41)	0.590
	Codominant	TT	47 (33.6%)	64 (46.7%)	1	0.090	Zhuang ethnic	21 (34.4%)	29 (45.3%)	1	0.430
		TC	71 (50.7%)	57 (41.6%)	0.59 (0.36–0.99)			32 (52.5%)	28 (43.8%)	0.61 (0.28–1.32)	
		CC	22 (15.7%)	16 (11.7%)	0.55 (0.26–1.16)			8 (13.1%)	7 (10.9%)	0.66 (0.21–2.12)	
	Dominant	TT	47 (33.6%)	64 (46.7%)	1	0.029		21 (34.4%)	29 (45.3%)	1	0.200
		TC-CC	93 (66.4%)	73 (53.3%)	0.58 (0.36–0.95)			40 (65.6%)	35 (54.7%)	0.62 (0.30–1.29)	
	Log-additive	---	---	---	0.75 (0.43–1.28)	0.280		---	---	0.75 (0.43–1.28)	0.280
MPA	Codominant	TT	61 (32.8%)	86 (46.2%)	1	0.027	Not MPA	7 (46.7%)	7 (46.7%)	1	0.82
		TC	97 (52.1%)	79 (42.5%)	0.57 (0.37–0.89)			5 (33.3%)	6 (40.0%)	0.99 (0.19–5.31)	
		CC	28 (15.1%)	21 (11.3%)	0.53 (0.28–1.02)			3 (20.0%)	2 (13.3%)	0.48 (0.04–5.59)	
	Dominant	TT	61 (32.8%)	86 (46.2%)	1	0.0075		7 (46.7%)	7 (46.7%)	1	0.83
		TC-CC	125 (67.2%)	100 (53.8%)	0.56 (0.37–0.86)			8 (53.3%)	8 (53.3%)	0.84 (0.17–4.07)	
	Log-additive	---	---	---	0.68 (0.50–0.93)	0.014		---	---	0.76 (0.24–2.38)	0.64

**Table 5:** Analysis of *TNFSF13B/BAFF* gene haplotype and the risk of AAV pathogenesis.

Haplotype	AAV (n = 201), n (%)	Control (n = 201), n (%)	OR (95% CI)	*p*
TA	193 (48.0)	186 (46.3)	1	0.621
CA	131 (32.6)	163 (40.5)	0.71 (0.53–0.95)	0.019
TT	78 (19.4)	53 (13.2)	1.59 (1.09–2.32)	0.017

**Table 6:** Association of *PADI4* gene polymorphisms with AAV susceptibility under different genetic models.

SNP	Model	Genotype	Control	AAV	OR (95% CI)	*p*
rs11203366	Codominant	AA	68 (33.8%)	67 (33.3%)	1	0.720
		GA	100 (49.8%)	95 (47.3%)	0.96 (0.62–1.49)	
	Dominant	GG	33 (16.4%)	39 (19.4%)	1.20 (0.68–2.13)	
		AA	68 (33.8%)	67 (33.3%)	1	0.920
		GA-GG	133 (66.2%)	134 (66.7%)	1.02 (0.67–1.55)	
rs874881	Log-additive	---	---	---	1.07 (0.81–1.42)	0.620
	Codominant	CC	67 (33.3%)	67 (33.3%)	1	0.53
		GC	101 (50.2%)	93 (46.3%)	0.92 (0.59–1.43)	
	Dominant	GG	33 (16.4%)	41 (20.4%)	1.25 (0.71–2.21)	
		CC	67 (33.3%)	67 (33.3%)	1	0.99
	Log-additive	GC-GG	134 (66.7%)	134 (66.7%)	1.00 (0.66–1.51)	0.570
		---	---	---	1.08 (0.82–1.43)	
SNP	Model	Genotype	Control	AAV	OR (95% CI)	*p*

**Table 7:** Comparison of clinical indicators among different genotypes of *TNFSF13B/BAFF* gene rs3759467 and rs1041569.

rs3759467					rs1041569			
Parameter	TT	TC	CC	*p*	AA	TA	TT	*p*
	(n = 139)	(n = 147)	(n = 38)		(n = 218)	(n = 95)	(n = 11)	
Rash (present/absent)	5/134	11/136	9/29	<0.001	23/195	2/93	0/11	0.023
Hemoptysis (present/absent)	17/122	16/131	11/27	0.012	37/181	7/88	0/11	0.03
BVAS	17.00 (13.00, 20.00)	17.00 (14.00, 20.00)	17.00 (12.75, 19.25)	0.643	17.00 (14.00, 20.25)	16.00 (14.00, 20.00)	17.00 (12.00, 19.00)	0.321
**rs3759467**					**rs1041569**			
Parameter	TT	TC	CC	*p*	AA	TA	TT	*p*
	(n = 139)	(n = 147)	(n = 38)		(n = 218)	(n = 95)	(n = 11)	
WBC	7.55 (5.63, 10.55)	8.16 (5.92, 10.89)	6.77 (5.42, 9.26)	0.263	7.76 (5.70, 10.70)	7.76 (5.80, 9.60)	7.40 (4.12, 8.97)	0.519
RBC	2.74 (2.33, 3.67)	2.90 (2.34, 3.64)	2.88 (2.20, 3.60)	0.758	2.90 (2.31, 3.73)	2.77 (2.41, 3.61)	2.45 (1.98, 2.61)	0.022
HB	79.00 (66.50, 104.00)	81.60 (64.60, 99.00)	73.00 (62.18, 88.25)	0.456	78.00 (63.78, 103.20)	80.20 (67.00, 97.00)	61.00 (44.00, 82.00)	0.032
PLT (platelet, PLT)	249.30 (184.00, 333.60)	265.10 (192.00, 354.00)	279.00 (207.00, 418.33)	0.119	266.55 (194.75, 369.50)	249.00 (182.00, 336.80)	252.00 (194.90, 323.50)	0.624
UALB (urinary albumin, UALB)	952.10 (299.00, 2129.90)	811.600 (373.00, 1736.80)	1037.80 (597.50, 1668.60)	0.771	927.18 (406.35, 1839.95)	785.00 (267.90, 1834.60)	1618.00 (223.30, 3908.00)	0.383
Scr (umol/L)	343.00 (122.00, 582.00)	369.00 (152.00, 522.00)	304.00 (108.00, 664.25)	0.974	337.00 (122.75, 524.00)	343.00 (112.00, 591.00)	506.00 (323.00, 1029.00)	0.127
CRP (mg/L)	15.00 (5.20, 53.79)	19.80 (5.70, 62.00)	14.62 (5.58, 34.86)	0.468	19.35 (5.72, 58.96)	12.00 (5.21, 47.69)	16.50 (3.02, 23.50)	0.322

**Table 8:** Association of *PADI4* gene rs11203366 and rs874881 genotypes with laboratory examination parameters.

rs11203366					rs874881			
Parameter	GG	GA	AA	*p*	GG	GC	CC	*p*
	(n = 69)	(n = 148)	(n = 107)		(n = 71)	(n = 145)	(n = 107)	
Rash (present/absent)	6/63	11/137	8/99	0.943	6/65	11/134	8/99	0.968
Hemoptysis (present/absent)	9/60	20/128	15/92	0.983	10/61	19/126	15/92	0.970
BVAS	17.00 (13.50, 19.50)	16.00 (13.00, 20.00)	18.00 (15.00, 21.00)	0.029	17.00 (14.00, 19.00)	16.00 (13.00, 20.00)	18.00 (15.00, 21.00)	0.032
WBC	7.84 (5.37, 10.63)	7.76 (5.96, 10.70)	7.48 (5.63, 10.03)	0.499	7.84 (5.37, 10.55)	7.76 (5.99, 10.73)	7.48 (5.63, 10.03)	0.415
RBC	2.74 (2.29, 3.60)	3.14 (2.57, 3.77)	2.65 (2.15, 3.25)	<0.001	2.74 (2.28, 3.55)	3.14 (2.58, 3.83)	2.65 (2.15, 3.25)	<0.001
HB	75.00 (63.00, 98.70)	86.00 (70.00, 107.00)	73.00 (60.00, 86.00)	<0.001	75.00 (63.00, 98.40)	86.00 (70.50, 107.35)	73.00 (60.00, 86.00)	<0.001
PLT (platelet, PLT)	237.00 (191.50, 328.80)	266.10 (198.00, 344.00)	265.00 (186.00, 390.00)	0.403	249.00 (192.00, 341.40)	263.00 (197.00, 337.90)	265.00 (186.00, 390.00)	0.603
UALB (urinary albumin, UALB)	785.00 (332.84, 1796.25)	783.00 (296.60, 1799.80)	1203.60 (552.00, 2274.40)	0.099	785.00 (298.78, 1810.50)	783.00 (317.30, 1741.45)	1203.60 (552.00, 2274.40)	0.102
Scr (umol/L)	341.00 (136.50, 547.50)	341.00 (118.00, 516.00)	384.00 (143.00, 688.00)	0.407	341.00 (125.00, 531.00)	341.00 (118.00, 515.00)	384.00 (143.00, 688.00)	0.437
CRP (mg/L)	18.27 (6.25, 53.65)	15.20 (5.73, 47.69)	19.10 (5.21, 61.28)	0.828	18.70 (6.80, 53.50)	15.10 (5.61, 47.69)	19.10 (5.21, 61.28)	0.834

**Table 9:** (**a**) Performance averages of various models for *TNFSF13B/BAFF* rs3759467. (**b**) Average performance values of each model for the *TNFSF13B/BAFF* genotype rs1041569. (**c**) Average performance of various models for the *PADI4* gene rs11203366. (**d**) Average performance of each model for the *PADI4* gene rs874881.

**(a)**					
**Model**	**ACC**	**PRE**	**REC**	**F1-Score**	**AUC**
Logistic	0.5323	0.5389	0.4288	0.476	0.5361
SVM_Linear	0.5324	0.5427	0.4289	0.4781	0.5274
RandomForest	0.5324	0.5406	0.4292	0.4775	0.5247
XGBoost	0.5138	0.5117	0.5025	0.4925	0.5362
**(b)**					
**Model**	**ACC**	**PRE**	**REC**	**F1-Score**	**AUC**
Logistic	0.5277	0.5581	0.3025	0.3914	0.5308
SVM_Linear	0.5248	0.5435	0.3088	0.3901	0.5213
RandomForest	0.5231	0.5386	0.3055	0.3887	0.5304
XGBoost	0.5277	0.554	0.3053	0.3911	0.5349
**(c)**					
**Model**	**ACC**	**PRE**	**REC**	**F1-Score**	**AUC**
Logistic	0.4814	0.4857	0.4018	0.4225	0.5039
SVM_Linear	0.4907	0.4999	0.5984	0.5096	0.505
RandomForest	0.4922	0.4935	0.3552	0.3943	0.5206
XGBoost	0.5138	0.5118	0.4254	0.4471	0.5225
**(d)**					
**Model**	**ACC**	**PRE**	**REC**	**F1-Score**	**AUC**
Logistic	0.5093	0.5375	0.4129	0.4413	0.5044
SVM_Linear	0.4892	0.4986	0.3524	0.3907	0.5299
RandomForest	0.506	0.5099	0.386	0.4186	0.5357
XGBoost	0.4999	0.5004	0.5614	0.5054	0.5395

## Data Availability

The data supporting the findings of this study are available from the corresponding author upon reasonable request. Due to ethical and privacy restrictions (participant consent did not include public deposition of individual-level genetic and clinical data), the genotype and phenotype data are not deposited in a public repository but can be shared with qualified researchers after approval from the institutional ethics committee.

## Supplementary Materials

The following supporting information can be downloaded at: https://www.mdpi.com/article/10.3390/genes17060710/s1, Table S1. Functional annotation and selection rationale of candidate SNPs in *TNFSF13B/BAFF* and *PADI4*. Supplementary Table S2. *PADI4* rs11203366 Subgroup analysis of the relationship between polymorphism and AAV disease risk Supplementary Table S3. *PADI4* rs874881 Subgroup analysis of the relationship between polymorphism and AAV disease risk.

## Abbreviations

The following abbreviations are used in this manuscript:

BAFF	B-cell-activating factor
PADI4	Peptidylarginine deiminase 4
AAV	ANCA-associated vasculitis
SNPs	Single-nucleotide polymorphisms
NET	Neutrophil extracellular trap
CRP	C-reactive protein
PCR	Polymerase chain reaction
PSM	Propensity score matching
ORs	Odds ratios
LD	Linkage disequilibrium
PLT	Platelet
UALB	Urinary albumin, UALB
HB	Hemoglobin
RBC	Red blood cell
BVAS	Birmingham Vasculitis Activity Score
