# Supplementary material for: Genetic Association and Clinical Relevance of TNFSF13B/BAFF and PADI4 Polymorphisms in ANCA-Associated Vasculitis: A Case–Control Study with Genetic Model Analysis in Guangxi Population
Source: Genes (Basel). 2026 Jun 20;17(6):710. doi: 10.3390/genes17060710 (PMC13300086; doi:10.3390/genes17060710)
Supplement: Supplementary file 1 [file genes-17-00710-s001.zip › genes-4347037-supplementary.pdf]

Table S1. Functional annotation and selection rationale of candidate SNPs in *TNFSF13B/BAFF* and *PADI4*

Gene	SNP	GRCh38 position	Variant type / functional annotation	East Asian MAF	HWE p-value in controls	Selection rationale
TNFSF13B/BAFF	rs3759467 (formerly rs386492354)	chr13:108,267,192	SNV located within the TNFSF13B locus; no definitive coding consequence reported in dbSNP	0.294	0.373	Common East Asian candidate SNP in the TNFSF13B/BAFF pathway; rs386492354 was merged into rs3759467 in dbSNP Build 142
TNFSF13B/BAFF	rs1041569	chr13:108,267,195	SNV within the TNFSF13B locus; candidate regulatory-region variant	0.110	0.755	Previously investigated in autoimmune diseases; selected for BAFF-pathway relevance and adequate East Asian frequency
PADI4	rs11203366	chr1:17,331,039	Coding missense variant, p.Gly55Ser	0.4006	0.711	Common East Asian PADI4 missense variant previously studied in autoimmune diseases and PADI4 haplotypes
PADI4	rs874881	chr1:17,334,004	Coding missense variant, p.Gly112Ala	0.4087	0.624	Common East Asian PADI4 missense variant previously studied in autoimmune diseases and PADI4 haplotypes

Supplementary Table S2 *PADI4* rs11203366 Subgroup analysis of the relationship between polymorphism and AAV disease risk

Group	Model	Genotype	Control	AAV	OR (95%CI)	p	Group	Control	AAV	OR (95%CI)	p
Female	Codominant	AA	44(35.8%)	38(32.2%)	1	0.470	Male	24(30.8%)	29(34.9%)	1	0.810
		GA	59(48%)	54(45.8%)	1.00(0.56-1.78)			41(52.8%)	41(49.4%)	0.80(0.40-1.61)	
		GG	20(16.3%)	26(22%)	1.51(0.73-3.14)			13(16.7%)	13(15.7%)	0.83(0.32-2.14)	
	Dominant	AA	44(35.8%)	38(32.2%)	1.OO	0.660		24(30.8%)	29(34.9%)	1	0.520
		GA-GG	79(64.2%)	80(67.8%)	1.13(0.66-1.94)			54(69.2%)	54(65.1%)	0.81(0.41-1.57)	
	Log-additive	---	---	---	1.19(0.84-1.71)	0.330		---	---	0.89(0.56-1.41)	0.610
Han ethnic	Codominant	AA	47(33.6%)	46(33.6%)	1	0.740	Zhuang ethnic	21(34.4%)	21(32.8%)	1	0.890
		GA	71(50.7%)	65(47.5%)	0.94(0.56-1.60)			29(47.5%)	30(46.9%)	1.00(0.45-2.23)	
		GG	22(15.7%)	26(19%)	1.23(0.61-2.47)			11(18%)	13(20.3%)	1.26(0.45-3.49)	
	Dominant	AA	47(33.6%)	46(33.6%)	1.OO	0.970		21(34.4%)	21(32.8%)	1	0.860
		GA-GG	93(66.4%)	91(66.4%)	1.01(0.61-1.67)			40(65.6%)	43(67.2%)	1.07(0.50-2.26)	
	Log-additive	---	---	---	1.08(0.77-1.52)	0.660		---	---	1.10(0.67-1.82)	0.700
MPA	Codominant	AA	66(35.5%)	63(33.9%)	1	0.890	Not MPA	3(20%)	4(26.7%)	1	0.240
		GA	89(47.9%)	89(47.9%)	1.04(0.66-1.64)			10(66.7%)	6(40%)	0.32(0.04-2.35)	
		GG	31(16.7%)	34(18.3%)	1.16(0.64-2.10)			2(13.3%)	5(33.3%)	1.58(0.15-16.15)	
	Dominant	AA	66(35.5%)	63(33.9%)	1	0.760		3(20%)	4(26.7%)	1	0.510
		GA-GG	120(64.5%)	123(66.1%)	1.07(0.70-1.64)			12(80%)	11(73.3%)	0.55(0.09-3.36)	

	Log-additive	---	---	---	1.07(0.80-1.43)	0.650		---	---	1.21(0.40-3.63)	0.740

Supplementary Table S3 *PADI4* rs874881 Subgroup analysis of the relationship between polymorphism and AAV disease risk

Group	Model	Genotype	Control	AAV	OR (95%CI)	p	Group	Control	AAV	OR (95%CI)	p
Female	Codominant	CC	43(35%)	38(32.2%)	1	0.280	Male	24(30.8%)	29(34.9%)	1	0.810
		GC	60(48.8%)	52(44.1%)	0.92(0.51-1.64)			41(52.8%)	41(49.4%)	0.80(0.40-1.61)	
		GG	20(16.3%)	28(23.7%)	1.60(0.77-3.31)			13(16.7%)	13(15.7%)	0.83(0.32-2.14)	
	Dominant	CC	43(35%)	38(32.2%)	1.OO	0.760		24(30.8%)	29(34.9%)	1	0.520
		GC-GG	80(65%)	80(67.8%)	1.09(0.63-1.86)			54(69.2%)	54(65.1%)	0.81(0.41-1.57)	
	Log-additive	---	---	---	1.21(0.85-1.73)	0.280		---	---	0.89(0.56-1.41)	0.610
Han ethnic	Codominant	CC	47(33.6%)	46(33.6%)	1	0.540	Zhuang ethnic	20(32.8%)	21(32.8%)	1	0.870
		GC	71(50.7%)	63(46%)	0.91(0.54-1.56)			30(49.2%)	30(46.9%)	0.92(0.41-2.05)	
		GG	22(15.7%)	28(20.4%)	1.32(0.66-2.64)			11(18%)	13(20.3%)	1.19(0.43-3.33)	
	Dominant	CC	47(33.6%)	46(33.6%)	1.OO	0.970		20(32.8%)	21(32.8%)	1	0.970
		GC-GG	93(66.4%)	91(66.4%)	1.01(0.61-1.67)			41(67.2%)	43(67.2%)	0.99(0.46-2.10)	
	Log-additive	---	---	---	1.11(0.79-1.55)	0.550		---	---	1.07(0.65-1.76)	0.800
MPA	Codominant	CC	65(35.0%)	63(33.9%)	1	0.770	Not MPA	3(20.0%)	4(26.7%)	1	0.240
		GC	90(48.4%)	87(46.8%)	0.99(0.63-1.56)			10(66.7%)	6(40.0%)	0.32(0.04-2.35)	
		GG	31(16.7%)	36(19.4%)	1.21(0.67-2.19)			2(13.3%)	5(33.3%)	1.58(0.15-16.15)	
	Dominant	CC	65(35.0%)	63(33.9%)	1	0.850		3(20.0%)	4(26.7%)	1	0.510
		GC-GG	121(65.0%)	123(66.1%)	1.04(0.68-1.60)			12(80.0%)	11(73.3%)	0.55(0.09-3.36)	
	Log-additive	---	---	---	1.08(0.81-1.44)	0.600		---	---	1.21(0.40-3.63)	0.740
